# *Pseudomonas aeruginosa* PAO 1 In Vitro Time–Kill Kinetics Using Single Phages and Phage Formulations—Modulating Death, Adaptation, and Resistance

**DOI:** 10.3390/antibiotics10070877

**Published:** 2021-07-19

**Authors:** Ana Mafalda Pinto, Alberta Faustino, Lorenzo M. Pastrana, Manuel Bañobre-López, Sanna Sillankorva

**Affiliations:** 1 INL-International Iberian Nanotechnology Laboratory, Av. Mestre José Veiga, 4715-330 Braga, Portugal; mafalda.pinto@inl.int (A.M.P.); lorenzo.pastrana@inl.int (L.M.P.); manuel.banobre@inl.int (M.B.-L.); 2 Centre of Biological Engineering, LIBRO—Laboratório de Investigação em Biofilmes Rosário Oliveira, University of Minho, 4710-057 Braga, Portugal; 3 Clinical Pathology Department, Hospital de Braga, 4710-243 Braga, Portugal; alberta.faustino@gmail.com

**Keywords:** *Pseudomonas aeruginosa*, bacteriophages, time–kill, motility, resistance

## Abstract

*Pseudomonas aeruginosa* is responsible for nosocomial and chronic infections in healthcare settings. The major challenge in treating *P. aeruginosa*-related diseases is its remarkable capacity for antibiotic resistance development. Bacteriophage (phage) therapy is regarded as a possible alternative that has, for years, attracted attention for fighting multidrug-resistant infections. In this work, we characterized five phages showing different lytic spectrums towards clinical isolates. Two of these phages were isolated from the Russian Microgen Sextaphage formulation and belong to the Phikmvviruses, while three Pbunaviruses were isolated from sewage. Different phage formulations for the treatment of *P. aeruginosa* PAO1 resulted in diversified time–kill outcomes. The best result was obtained with a formulation with all phages, prompting a lower frequency of resistant variants and considerable alterations in cell motility, resulting in a loss of 73.7% in swimming motility and a 79% change in swarming motility. These alterations diminished the virulence of the phage-resisting phenotypes but promoted their growth since most became insensitive to a single or even all phages. However, not all combinations drove to enhanced cell killings due to the competition and loss of receptors. This study highlights that more caution is needed when developing cocktail formulations to maximize phage therapy efficacy. Selecting phages for formulations should consider the emergence of phage-resistant bacteria and whether the formulations are intended for short-term or extended antibacterial application.

## 1. Introduction

*Pseudomonas aeruginosa* is an opportunistic pathogen and a leading cause of severe nosocomial infections [1,2]. *P. aeruginosa* infections in healthcare settings include pneumonia, surgical, wound, and urinary tract infections and are associated with risk factors such as mechanical ventilation, immunosuppression, catheterization [2,3,4,5]. Effective treatment of *P. aeruginosa* infections is challenging [6], and multidrug-resistant and extensively drug-resistant *P. aeruginosa* have increased in prevalence and augmented morbidity and mortality [2,7,8]. *P. aeruginosa* is listed since 2017 as one of the critical priority pathogens listed by the World Health Organization to encourage research and development into new antibacterials [9].

Bacteriophage (phage) therapy is in use under the umbrella of article §37 (Unproven Interventions in Clinical Practice) of the Helsinki Declaration of Ethical Principles for Medical Research Involving Human Subjects [10,11]. In addition, the compassionate treatment of patients using phages is set out by different regulatory agencies, such as the Food and Drug Administration and the European Medicines Agency [12]. Commercial phage formulations are available in Russian and Georgian pharmacies and target a particular species or a panoply of species. *P. aeruginosa* phages are present in the Pyo- and Intesti-bacteriophage (Eliava BioPreparations, <10^5^ plaque forming units (PFU)/mL), and in the complex Pyobacteriophage, Sextaphage, and Intesti-bacteriophage preparations (Microgen, at unspecified concentrations). Although each formulation target is acknowledged, the number of phages for each is not detailed. Metagenomics of Russian and Georgian pyophage cocktails evidenced the existence of 41 full-length phage genomes. In both formulations, *P. aeruginosa* phages shared homology with the Luz24virus, but diverged in the other contributing phages [13]. Three PYO phage cocktails, produced from 1997 to 2014, included 30 (PYO 1997) and 29 (PYO 2000) draft genomes [14]. Only 11 were common in all preparations, evidencing that the cocktail formulations are not stagnant through times but instead tweaked to fit the predominant strains isolated. 

In this work, we fully characterized five *P. aeruginosa* PAO1 phages and evaluated the antimicrobial efficacy of different formulations, assessing their impact on killing, the emergence of insensitive mutants, and evaluated the motility and virulence changes.

## 2. Results

### 2.1. Selection of Phages Based on Host Range

In this work, we tested 30 phages against *P. aeruginosa* PAO1 and clinical isolates. Isolates were collected from hospitalized patients with different infections. The patients were aged from 3 (male, ear infection) to 90 (female, urinary tract infection) years old. The majority were isolated from blood (42.1%), followed by urine (26.3%) and sputum (21.1%). The antimicrobial susceptibility of the clinical isolates varied from sensitive to all antibiotics (I500546) up to resistant to six antibiotics (I93488) (Table 1). All isolates were sensitive to aztreonam, cefepime, colistin sulfate, and ticarcilin with clavulanic acid. In addition, maximum resistance was observed for seven different isolates to gentamicin and ciprofloxacin.

Phages’ lytic spectra varied from 15 to 55%. Lysis from without was perceived following tests in two urine isolates (U572569, I97824) and one blood isolate (I60026) (Table 2). In these clinical isolates, lysis occurred due to the interaction of multiple phages with the outer membrane of the isolates and not due to a lytic infection cycle and consequent release of progeny phages. Four clinical isolates (I499131, I29074, I41151, and U14706) were utterly insensitive to all phages (Table 2). These four did not share similarities in antibiotic susceptibilities changing in resistance towards one antibiotic (aminoglycosides) up to four antibiotic classes (isolate I41151) (Table 1).

Phages were selected were based on differences in the spectrum of activity. Phages SPCB and SPCG, included in the Sextaphage formulation, lysed 50 and 55% of the tested isolates. The spectra showed SPCB’s ability to infect C364224, I60026, and I60584, and SPCG’s killing isolates I97824 and C80117. SMS12, SMS21, and SMS29 isolated from raw sewage, lysed between 30 and 45%. SMS12 infected U570696 that both Sextaphages did not lyse. SMS21 also killed this isolate and further lysed H73832; however, it could not infect I202628.

### 2.2. Virion Particle and Plaque Morphologies

**Figure 1:**
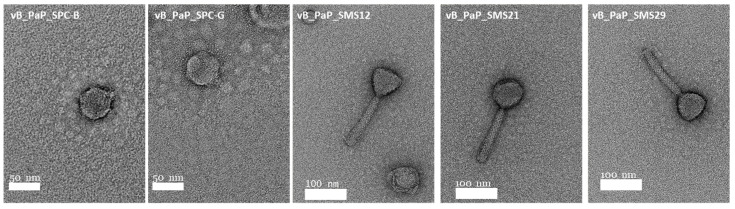
Virion particle morphology of *P. aeruginosa* PAO1 phages observed by TEM.

The characteristics of two phages previously isolated [15] and three additional phages were studied in terms of virion and plaque morphologies (Figure 1). Phages SPCB and SPCG had short tails resembling members of the *Autographiviridae* family. On the other hand, phages SMS12, SMS21, and SMS29 had long contractile tails resembling phages of the *Myoviridae* family.

**Figure 2:**
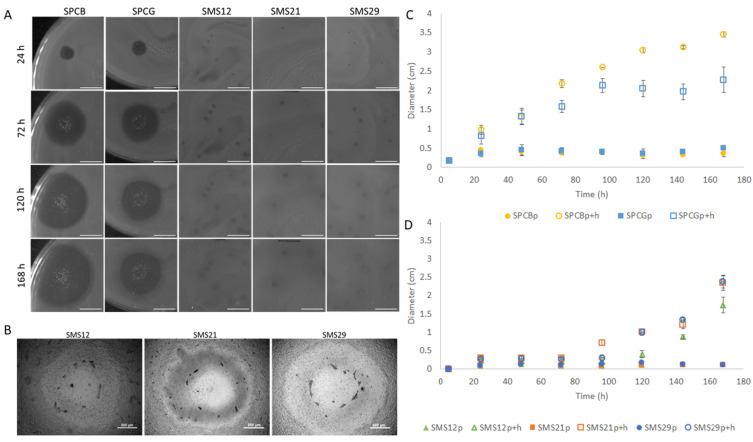
Plaque characteristics of the five phages used in this work after 24, 72, 120, and 168 h: (**A**) morphological characteristics (scale bar 1 cm); (**B**) microscopy of phages SMS12, SMS21, and SMS29 (5× objective) (scale bar 500 µm); (**C**) plaque diameters of phages SPCB and SPCG); (**D**) plaque diameters of phages SMS12, SMS21, and SMS2. Data in (**C**) and (**D**) are shown either as mean of plaque diameter (p) ± SD or mean of plaque and halo diameter (p + h) ± SD.

Plaque morphologies varied from tiny (1 mm) to big (5 mm), and the plaque + halo (p + h) diameters ranged between 3 and 35 mm (Figure 2). SPCB and SPCG had considerably larger plaques (p) which did not alter with time, and haloes that increased, since the start (Figure 2A), at average speeds of 0.41 ± 0.24 and 0.43 ± 0.18 cm/day until 168 h, respectively (Figure 2C). In addition, the lysis zones of SPCB and SPCG had significant numbers of colonies already after 24 h. The haloes of SMS12, SMS21, and SMS29 were smaller (p + h, Figure 2B) and started to increase after 96 h at average speeds of 0.51 ± 0.33, 0.51 ± 0.48, and 0.69 ± 0.35 cm/day (Figure 2D), respectively.

### 2.3. One-Step Growth Characteristics

**Figure 3:**
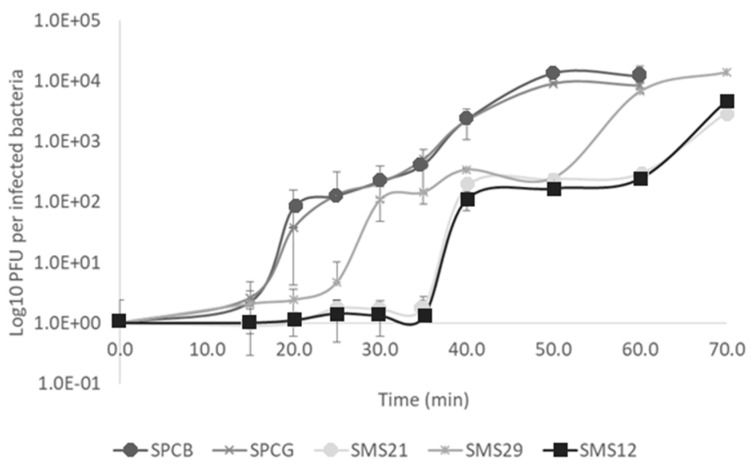
One-step growth characteristics of the five phages used. Data are shown as mean ± SD.

One-step growth experiments with the five phages were performed (Figure 3).

Phages SPCB and SPCG reached 141.5 ± 70.5 and 169.0 ± 52.9 PFU/infected bacteria having similar latent periods (15 min). The other three phages resulted in burst sizes of 138.5 ± 40.3 (SMS12), 209.0 ± 41.0 (SMS21), and 199.0 ± 25.5 (SMS29) PFU/infected bacteria, respectively. Despite this resemblance in burst size, SMS29 presented a 10 min shorter latent period than SMS12 and SMS21 (Figure 3).

### 2.4. Phage Genomes and Comparative Analysis

**Figure 4:**
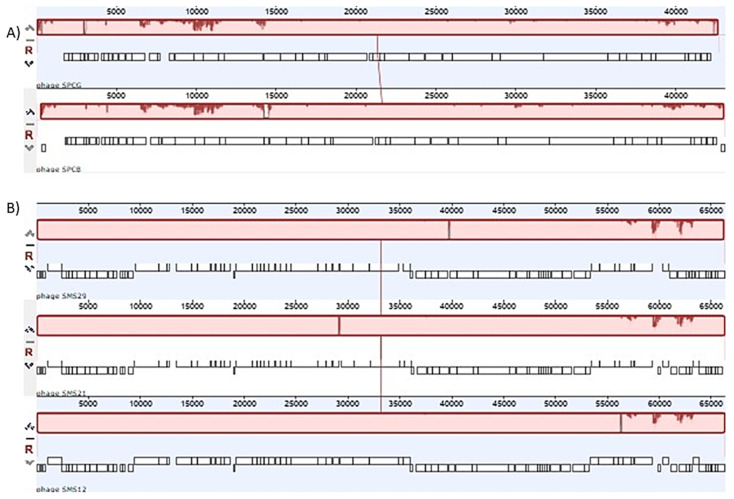
Comparison of phages from the two families: (**A**) Progressive MAUVE genome alignment between phages SPCB (above) and SPCG (below); (**B**) Progressive MAUVE genome alignment between phages SMS12, SMS21, and SMS29. White gap areas show regions that were not aligned and contain sequence elements specific to a particular genome. The height of the similarity profile presents an average level of conservation in a particular region of the genome sequence.

SPCB and SPCG resembled Phikmvviruses from the *Krylovirinae* sub-family of the *Autographiviridae* family. Pairwise identity at the nucleotide level was 97.1%, with minor differences in the regions between ORFs 4–6 of SPCG and ORF 25 of SPCB (Figure 4A, Table S1). In addition, both SPCB and SPCG showed homology to phages PT5 (EU056923) and vB_Pae_TbilisiM32 (NC_017865) but at different percentages (Supplementary Tables S2 and S3 and Supplementary Figures S1 and S2).

Pairwise identity, at the nucleotide level, between SMS12, SMS21, and SMS29, was above 98%, with only a few unique regions (Figure 4B, Table S1). The most considerable differences started following the DNA primase (ORF_0074 in phages SMS21 and SMS2, ORF_0075 in SMS12) until ORF_0082. All three phages resembled Pbunaviruses, more specifically JG024 (NC_017674, and GU815091), phage 14-1 (NC_011703), respectively (Supplementary Tables S4–S6 and Supplementary Figures S3–S5).

No transmembrane domains and no tRNAs were present in the genomes, indicating their sole dependence on the host tRNA molecules. Promoter numbers varied between 1 and 3. Rho-independent terminators found no terminators for SPCB, one for SPCG, eight for SMS12, seven for SMS21, and six for SMS29, respectively.

### 2.5. Time–Kill of Single Phage and Phage Cocktail Formulations

**Figure 5:**
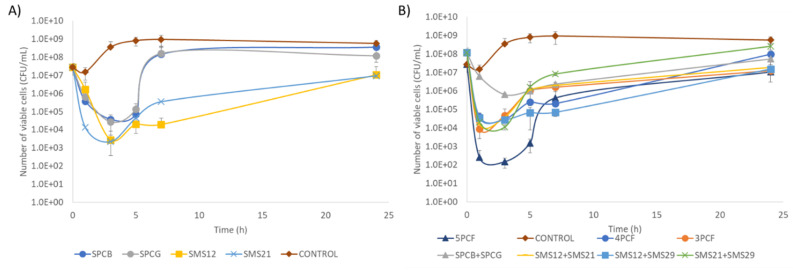
Time–kill experiment of each phage alone or in cocktail against *P. aeruginosa* PAO1. MOI of 10, 120 rpm, 37 °C: (**A**) phages alone; and (**B**) phages in different cocktail formulations. 5PCF (phages SPCB + SPCG + SMS12 + SMS21 + SMS29); 4PCF (SPCG + SMS12 + SMS21 + SMS29); 3PCF (SMS12 + SMS21 + SMS29). Data are shown as mean ± SD.

Time–kill experiments were performed with single phages or phage formulations (Figure 5A,B).

Time–kill experiments showed that Pbunaviruses (SMS12, SMS21, and SMS29) produced the best antibacterial effect (reducing approximately 4 log10 CFU/mL after 3 h). The Phikmvviruses SPCB and SPCG led to 3 log10 CFU/mL reductions (Figure 5A). After 3 h post-infection, a growth of *P. aeruginosa* cells was observed, which was faster for Phikmvviruses than Pbunaviruses. All phages, except SPCB, exhibited after 24 h post-infection significantly lower (*p* ≤ 0.05) viable cell counts compared to the controls.

The number of phages used in the multi-phage combination experiments (Figure 5B) varied from 2 to 5 phages. The five-phage cocktail formulation (5PCF) led to a faster lysis at 1 h (4.8 log10 CFU/mL reduction) that further increased after 3 h (6.4 log10 CFU/mL) (Figure 5B). Removing just phage SPCB from this 5PCF reduced the antibacterial efficacy of the formulation. In fact, this four-phage cocktail formulation (4PCF) (SPCG + SMS12 + SMS21 + SMS29), and the two-phage formulations SMS12 + SMS21, SMS12 + SMS29, and SMS21 + SMS29 showed similar CFU/mL reductions (*p* > 0.05), oscillating between 4.0 and 4.5 log10 at 3 h. The combination of the two Sextaphage Phikmvviruses caused the lowest reduction (2.7 log10 at 3 h). A statistically significant CFU/mL difference (*p* ≤ 0.05), after 24 h, was observed between the control and the following cocktails: the three-phage cocktail formulation (3PCF) (SMS12 + SMS21 + SMS29, 1.7 log10), SMS12 + SMS29 (1.6 log10), and SMS12 + SMS21 (1.5 log10). As in single-phage experiments, a rapid increase in cells after a few hours of treatment also took place in all combination assays.

Due to the increase in *P. aeruginosa* after being challenged following a single and a multi-phage approach, surviving cells were recovered post-infection. Their susceptibility to all phages and possible changes in motility were evaluated to understand this growth phenomenon.

### 2.6. Assessment of the Survivor’s Susceptibility and Motility

Survivors were isolated following 24 h post-infection and assessed for their susceptibility to all phages used in this study (Table 3). Surviving cells were challenged with all phages and grouped into 18 specific resistance patterns, where R stands for resistance and S for susceptible to a given phage (Table 3). Even though we registered different susceptibility patterns following phage treatment, some recovered survivors continued to show susceptibility to the phage used but had acquired resistance towards other phages (Table 3). These susceptible survivors are highlighted in green, and an example of this is what was observed with phage SMS12. For example, survivors of SMS12 treatment showing patterns 7, 8, 10, 12, and 15, continued to be susceptible to this phage but had become resistant to one (patterns 12 and 15), two (patterns 8 and 10), or three (pattern 7) phages, respectively. It is also worth mentioning that *P. aeruginosa* PAO1 is susceptible to all phages, and these replicate to produce progeny. However, after 24 h, colonies obtained from the control samples (not challenged with any phage) presented resistance towards phages SPCB and SPCG (patterns 15—SRSSS (30%), and 8—RRSSS (30%)).

A higher number of patterns showed that survivors had acquired resistance towards SPCB (79.6%) and SPCG (64.7%) (see the last two rows in the susceptibility profile columns). The 5PCF resulted in the highest number of patterns (nine patterns), followed by treatment with SPCB and SMS12 alone (six). Conversely, the least varying susceptibility profiles were achieved with individual phage treatments using SPCG, SMS21, and SMS29, and the two-phage SMS12 + SMS29 formulation. Although 5PCF presented the most patterns, the removal of a phage SPCB (4PCF) and the two Phikmvviruses phages (3PCF) increased the percentage of bacteria resistant to four (R4) and five (R5) phages. R4 phage survivors after 5PCF were 30% (sum of patterns 2, 3, 5, 9) while after 4PCF and 3PCF, these increased to 57.1% (4PCF, sum of patterns 2, 3, and 5), and 50% (3PCF, patterns 2 plus 5), respectively. Additionally, R5 phage survivors (pattern 1) increased from 10% in 5PCF to 14.3% (4PCF) and 25% (3PCF) (Table 3). The removal of all Pbunaviruses from the 5PCF increased R5 phage survivors from 10% (5PCF) to 33.3% (SPCB + SPCG), respectively.

The results between any two-phage formulation comprising two of the following phages: SMS12, SMS21, and SMS29, vary considerably compared to the 3PCF consisting of the same phages. For instance, in terms of R5, the two-phage formulations had either lower (7.5%, SMS12 + SMS21), equal (25%, SMS21 + SMS29), or higher percentages (80%, SMS21 + SMS29).

In addition to the susceptibilities of surviving cells towards the different phages, survivors were also evaluated for possible changes in motility (Table 4). Several motility differences were registered, particularly considering the predominant swimming and swarming motilities (blue and grey highlighted values). Although the control cells (non-phage challenged) showed changes in susceptibility to phages, 100% of the survivors maintained excellent swimming characteristics and dendritic swarming motility. Additionally, survivors from single SMS21 and SMS29 phage experiments remained mostly good swimmers (87.5 and 79%, respectively), while those following treatment with phages SPCB, SPCG, and SMS12 changed their swimming predominance to nonswimmers. Compared to the non-phage-treated survivors, changes in swarming were only perceived for single phage treatments with SMS12 and SMS29.

The use of phage cocktails also resulted in motility shifts compared to non-phage exposed *P. aeruginosa* PAO1 (Table 4). Only survivors from SPCB + SPCG remained good swimmers (50%), although in a fairly similar amount to nonswimmers (45.5%). Therefore, the other cocktail formulations will be compared, regarding swimming characteristics, only in terms of the percentage changes in nonswimming survivors. The 5PCF treatment resulted in 73.7% of nonswimmers and a predominant smooth edge swarming (79.0%). The removal of phage SPCB from the 5PCF decreased nonswimmers to 38.4% (4PCF). However, this removal increased nonswarmers from 15.8% (5PCF) to 57.1% (4PCF) (Table 4). The removal of both SPCB and SPCG from the 5PCF caused a decrease in nonswimmers (42.9%, 3PCF) and increased nonswarmers (87.5%, 3PCF).

Comparing the 3PCF (SMS12 + SMS21 + SMS29) survivors and any of the two-phage formulations prepared with these phages showed an increase in nonswimmers from 42.9% (3PCF) to 71.4% (SMS12 + SMS21), 55.6% (SMS12 + SMS29), and 60.0% (SMS21 + SMS29), respectively. Additionally, the predominant swarming motility changed considerably from dendritic (87.5%, 3PCF) to nonswarmers (SMS12 + SMS21, 71.4%), with a smooth edge (SMS12 + SMS29, 57.1%). Only SMS21 + SMS29 kept the same swarming motility as the 3PCF, although this was reduced from 87.5 to 50.0%.

**Figure 6:**
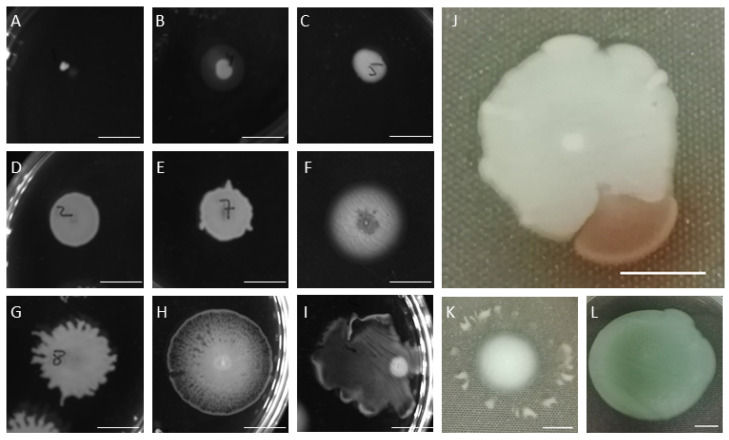
Different swimming motilities observed in surviving cells: (**A**–**I**) images acquired using Chemi XT4; and (**J**–**L**) digital photographs.

**Figure 7:**
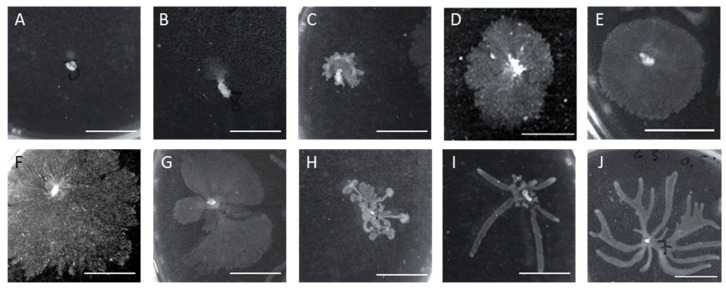
Different swarming motilities observed in surviving cells. (**A**–**J**) images acquired using Chemi XT4.

A few examples of swimming and swarming profiles are present in Figure 6 and Figure 7. In terms of swimming, some survivors had no motility (Figure 6A) while others presented reduced (Figure 6B,C), moderate (Figure 6D–F), and excellent swimming competencies (Figure 6G–I). A few survivors also produced flares that swam beyond the uniform swimming zone (Figure 6J,K), and a minor fraction presented, after 48 h, a red/brown pigmented area indicative of pyorubrin production (Figure 6I).

Differences in swarming behaviors were perceived, particularly in the swarm zone coverage, tendril formation, and alterations related to the swarm zone edges (Figure 7). Some survivors lost their swarming motility (Figure 7A). Furthermore, most cells presented smooth edges with wandering colonies (Figure 7D–F) or multiple fronts radiating outwards that swarmed fast due to the developed tendrils (Figure 7I,J). In addition, some survivors showed deep creases connected to the central swarm zone (Figure 7C,H), while others showed suppressors emerging from the central colony as motility flares (Figure 7B,G).

## 3. Discussion

The clinical use of phages has witnessed significant advances after several compassionate reports and the successful healing of antibiotic-resistant infections. However, despite the enthusiasm for phage therapy, phage treatments cause the emergence of phage-insensitive phenotypes, which can compromise the therapeutic outcome. A recent review showed that phage-insensitive variants occurred in 80% of studies targeting the intestinal milieu, 50% of studies using sepsis models, and 75% of humans [15]. 

This study focuses on the impact of different phage formulations on the killing, survival, and resistance of *P. aeruginosa*. *P. aeruginosa* is a member of the challenging ESKAPE pathogens group, which shows an excellent ability to “escape” killing by antibiotics [16,17,18]. The virulence of *P. aeruginosa* has been attributed to several cell-associated factors such as LPS, flagellum, as well as pilus and non-pilus adhesins, and to exoenzymes or different secretory virulence factors [19]. 

Five *P. aeruginosa* PAO1 phages were characterized and used alone and in combination to control this strain. The Phikmvviruses (SPCB and SPCG) showed high homology to many Phikmvviruses, with burst sizes (116 (phages phiKMV and PT2) to 225 (phage LKA1) PFU/infected cell), and genome lengths in agreement with those reported (41,593 bp (phage LKA1)–43,548 bp (LUZ19)) [20]. The three other phages were Myoviruses, resembling other Pbunaviruses. Their genome lengths agreed with other Pbunaviruses (64,423 bp for phage LBL3 up to 68,871 bp for phage vB_Pae_PS44). However, SMS21 and SMS29 had slightly superior burst sizes to those reported (64 (phage KT28) [21] to 180 (phage JG024) PFU/infected bacteria [20,22]), with 209 and 194 PFU/infected bacteria, while SMS12 presented a burst in that range (138 PFU/infected bacteria). In addition, SMS12 and SMS21 had latent periods of 35 min, which are typically observed with other Pbunaviruses (35 min (KTN6 and KT28) [23] up to 50 min (phage JG024) [22]). 

Single phage application significantly reduced the living cell population, and the best antibacterial efficacy was perceived 3 h post-treatment with the Pbunaviruses (Figure 5A). Despite Phikmvviruses’ (SPCB and SPCG) shorter latent period, this factor did not significantly outweigh the antibacterial performance of other phages. In fact, SPCB and SPCG caused the lowest viable cell reductions and the most rapid increase in survivors. On the other hand, Myoviruses use in the experiments maintained the viable cell counts low until 7 h of treatment.

In terms of combinatory phage experiments, only six phage combinations were tested (Figure 5B). The selection of the combinations tested relied on the limited antibacterial action of the dual combination of SPCB + SPCG. Different phage formulations gave rise to startling results at the population level. Cell death was much faster after one hour and may hypothetically be due: (i) to lysis-from-without phenomena; or (ii) cooperation between *Autographiviridae* and *Myoviridae*, which use different host cell receptors for adsorption (discussed below). Lysis was not further enhanced between 1 and 3 h of treatment (Figure 5B), as had previously occurred in single-phage treatments (Figure 5A). The loss of further activity may be due to competition between the access of the phages to the host receptors. Although the multiplicity of infection remains the same, taking into account each phage and number of bacteria present in the culture, the combination of phages in a cocktail can double the number of phages targeting a specific receptor when the two Phikmvviruses or two of the three Pbunaviruses are combined or even triple the phages available for adsorption to a receptor when SMS12, SMS21, and SMS29 are combined. Competition for receptors in the cell wall delays adsorption and concomitantly delays phage lysis [24,25], as recently demonstrated using fluorescently labeled phages [24]. In that work, the authors showed observed that the lysis only derived due to one phage relative to the mixed lysis fluorescence, suggesting a direct or indirect suppression of one of the phages at some point. Phage dominance was due to the blocking of DNA replication through resource sequestration, and the impotence of phage was due to an incomplete ejection of the DNA into the cell.

The selective pressure from phages on their host population can result in potential alterations in phage receptors that may hinder the phage adsorption step [26,27] and change the virulence of the emerged variants [28]. Many have reported lower frequencies of phage-resistant mutants using cocktail formulations rather than monotherapy against *Klebsiella pneumoniae* [29] and *Escherichia coli* [30,31]. The results presented herein are in agreement with these articles. The 4PCF and 3PCF gave higher percentages of phage-resistant bacteria compared to 5PCF. Nevertheless, this was not universal, since this hypothesis did not apply to all two-phage formulations that were prepared using the same phages used to produce the 3PCF.

Survivors from control experiments became insensitive to two phages, but this can be due to the phenotypic variations of *P. aeruginosa* PAO1 itself that are known to occur at a relatively high frequency [32].

Phages can use different host receptors for adsorption, such as regions of the LPS (O-antigen (phage P22 [33]), outer core (phage SSU5 [34])); outer membrane proteins (e.g., FepA and TonB (phage H8 [35]), OmpC (phage T4 [36])); type IV pili (phages DLP1 and DLP2 [37]), flagella (phage 7-7-1 [38]); among others. Phages related to phiKMV, such as SPCB and SPCG, are pili-dependent [20,39], and Pbunaviruses, such as phages SMS12, SMS21, and SMS29 are LPS-dependent [39].

*P. aeruginosa* possesses two surface structures, a single polar flagellum (flagella) and a polar type IV pili (TFP), that facilitate its motility [40], and both can serve as phage receptors. Flagella are mostly virulence factors for the establishment, persistence, and inflammatory profile and a common cause of acute and chronic of *P. aeruginosa* infections [23]. Flagella are not permanent cellular structures; instead, the cell’s probability of having a flagellum differs across different growth phases [41]. Bacteria without flagella generally cause less inflammation and mortality [42,43]. The swimming motility of the survivors varied according to the phage(s) used, and higher losses in motility were achieved in treatments with the 5PCF and the SMS12 + SMS21 cocktails, presenting 73.7 and 71.4% of nonswimmers, respectively. In theory, these nonswimmers may be defective in flagella, and, as a positive consequence, these cells will have a decline in virulence and be less prone to form biofilms on surfaces and tissues.

The swarming of *P. aeruginosa* PAO1 is typically characterized by a dendritic colonial appearance. However, when changes in flagellar quantity and placement or both occur, swarming motility can be compromised. Individual Myoviruses, the 3PCF, and SMS12 + SMS21 revealed the highest losses in swarming motility. Diminished or absent swarming motility can result from a loss of the signal recognition particle-like protein FlhF, resulting in the assembly of flagella at nonpolar locations on the cell resulting in defective swimming and swarming motilities in *P. aeruginosa* [44]. Additionally, mutations in LPS can influence the exposition of flagella and pili on the bacterial surface, such as that observed with PAO1 ΔwaaL mutants which encoded a functional O-antigen ligase, which showed drastic alterations in swimming and twitching motilities [45,46]. This may justify the loss of infectivity of phages in some of these experiments performed with the Myoviruses.

## 4. Materials and Methods

### 4.1. Bacteria, Phages, Growth Conditions

This work used *P. aeruginosa* PAO1 (DSM 22644) and different clinical isolates (Table 1). All strains were grown at 37 °C in liquid tryptic soy broth (TSB) or solid TSB medium (TSB + 1.2% (*w*/*v*) of agar). The *Pseudomonas* phages used were isolated from the Sextaphage Cocktail (SPC) (Microgen, ImBio Nizhny Novgorod, Russia, series N789 date of issue 03/2016) [47] and also from raw sewage, as previously described [48].

### 4.2. Phage Host Range Determination

Phages were tested against a panel of isolates using the standard spot test, with phages being serially diluted in SM buffer (5.8 g/L NaCl, 2 g/L MgSO4.7H2O, and 50 mL/L of 50 mM Tris/HCl (pH 7.5)) to investigate lysis from within and from without phenomena [49].

### 4.3. Phage Propagation and Titration

Phage amplification was performed using the plate lysis and elution method [50], and phage titrations were performed according to a previously described method [51].

### 4.4. Phage Plaque Morphology and Replication Characteristics

Ten different plaques were analyzed in terms of plaque and halo widths using a high-performance imaging apparatus (Chemi XT4, GBOX-CHEMI-XT4-E, AlphaMetrix Biotech, Rödermark, Germany), coupled with a 4.2 MP imaging 16-bit CCD camera. Phage growth parameters were determined, as previously described [48]. In brief, for single-step experiments, 10 mL of a mid-exponential-phase culture was harvested by centrifugation (7000× *g*, 5 min, 4 °C) and the pellet resuspended in 5 mL fresh TSB (OD_600_ of 1.0). Phages (5 mL) were added at a MOI of 0.001, homogenized and allowed to adsorb for 5 min at room temperature. The samples were centrifuged (7000× *g*, 5 min, 4 °C) and the pellet resuspended in 10 mL of fresh TSB. Samples were taken during a period of 70 min. The samples were serially 10-fold diluted and plated immediately. Plaque forming units (PFU) were determined following 16 h incubation at 37 °C.

### 4.5. Transmission Electron Microscopy (TEM) Analysis of Phages

Phage particles were centrifuged for one hour (25,000× *g*, 4 °C) and the pellet was washed twice in tap water and centrifuged once more (1 h, 25,000× *g*, 4 °C). Phages were deposited on copper grids (400 mesh), stained with 2% (*w*/*v*) uranyl acetate (pH 4.0), and imaged at 200 kV using a JEM-2100-HT electron microscope (JEOL, Tokyo, Japan).

### 4.6. Phage DNA Extraction, Genome Sequencing, and Annotation

Phage DNA was extracted using the quick-DNA Viral kit (Zymo Research, D3015) according to the manufacturer’s specifications, and sequencing performed on an Illumina HiSeq platform after DNA library preparations using the Illumina Nextera XT library preparation kit. The reads obtained were trimmed to remove adapters, contaminations, or low-quality sequences. Contigs were assembled with PATRIC 3.5.43, running BayesHammer on reads, assembling with Velvet, IDBA and SPAdes, and sorting assemblies by ARAST quality score (https://www.patricbrc.org/, 15 September 2019) and manual inspection. Genomes were auto-annotated using MyRAST [52] and manually checked (Geneious Prime 2019, Biomatters, Newark, NJ, USA). Frameshifts were checked with BLASTX [53], and functions of translated open reading frames (ORFs) were searched using BLASTP [54] (E-value ≤ 10^–5^) and HHPRED server [55] (September–October 2019). Transmembrane domains were checked using TMHMM [56] and Phobius [57], tRNAs searched using tRNAscan-SE [58], and putative promoters using Promoter 2.0 [59]. ARNold was used to predict Rho-independent terminators [60], and the energy was calculated using Mfold [61]. Whole-genome comparisons using OrthoVenn [62] and Progressive MAUVE were performed [63]. The complete phage genome sequences were submitted to GenBank under the accession numbers: vB_PaeP_SPCB (MN615698); vB_PaeP_SPCG (MN615699); vB_PaeM_SMS12 (MN615700); vB_PaeM_SMS21 (MN615701); and vB_PaeM_SMS29 (MN615702).

### 4.7. Time–Kill Experiments with Different Formulations

The M26-A document of the Clinical & Laboratory Standards Institute was adopted to carry the time–kill experiments [64]. In brief, *P. aeruginosa* (1 mL, 5 × 10^8^ CFU/mL) was diluted in 9 mL of TSB, and 100 µL of phage (5 × 10^9^ PFU/mL) or 100 µL of SM buffer (control) were added. The mixtures were incubated at 37 °C (120 rpm), and samples were taken at 0, 1, 3, 5, 7, and 24 h post-infection. Serial 10-fold dilutions of *P. aeruginosa* cells were performed in saline containing 10 mM ferrous ammonium sulphate. Three independent experiments conducted in triplicate were performed. After 24 h of each independent time–kill experiment, 25 surviving colonies were isolated, and tested for their susceptibility not only to the phage they had been challenged with but also all other phages. The susceptibility assay was performed according to a previously described protocol [65].

### 4.8. Characterization of the Motility Properties of Survivor Cells

The swimming and swarming motilities of survivors were analyzed as previously described [66,67], and Petri dishes observed using Chemi XT4 coupled with a 4.2 MP imaging 16-bit CCD camera.

### 4.9. Statistical Analysis

Statistical analysis was performed using two-way ANOVA followed by Tukey’s multiple comparison statistical tests, using GraphPad Prism 6 (GraphPad Software, La Jolla, CA, USA). At least three independent experiments were performed, and the results are presented as mean ± standard deviation (SD). Differences were considered as statistically different if *p* ≤ 0.05 (95% confidence interval).

## 5. Conclusions

The results of this study show that universal assumptions regarding the decrease of phage-resistant variants using cocktails are not valid for any given phage combination. Many studies found in literature isolate phages, briefly characterize them, and combine a few in cocktails failing to properly identify the host receptors which they target. Therefore, caution is necessary when combining phages from the same genus due to potentially adverse outcomes. Ideally, the phage formulations should combine different genera, more than just the two used in this study, to better understand the phage-phage and phage-bacteria interactions and produce better antibacterial solutions than the commonly available ones. 

## Figures and Tables

**Table 1:** Antibiogram of *P. aeruginosa* clinical isolates used for assessing the lytic spectrum of phages.

ID	Source	Year	Sex (M/F) *	Age (Years)	Antibiotic													
					AK	TOB	CN	ATM	FEP	CAZ	CIP	CS	IMI	MRP	PRL	TZP	TC	TTC
I500546	Cutaneous	2013	M	82	8	≤1	≤1	8	≤2	4	0.25	≤1	2	2	8	≤2	8	4
I499131	Sputum	2013	F	59	>16	≤1	>4	8	≤2	4	0.25	≤1	2	4	4	≤2	8	4
C364224	Sputum	2013	M	52	4	≤1	≤1	8	≤2	4	2	≤1	2	2	8	≤2	8	≤2
U570696	Urine	2013	M	89	2	≤1	≤1	8	≤2	4	0.25	≤1	4	>16	8	8	8	4
U572569	Urine	2013	M	82	>16	>4	>4	8	≤2	4	4	≤1	2	>16	4	>32	8	≤2
I66897	Blood	2018	M	63	2	≤1	≤1	16	≤2	4	0.25	≤1	2	2	8	≤2	32	4
I29074	Blood	2017	M	68	2	≤1	≤1	16	≤2	16	1	≤1	2	2	8	32	8	8
I41151	Blood	2017	M	80	16	8	8	16	≤2	2	2	≤1	4	4	2	≤2	32	4
H73832	Blood	2018	M	61	2	≤1	≤1	16	≤2	4	0.25	≤1	2	4	4	≤2	>16	4
U88885	Blood	2017	F	74	4	≤1	≤1	16	≤2	2	0.25	≤1	4	2	4	≤2	8	4
I60026	Blood	2018	F	83	2	≤1	≤1	16	≤2	4	0.25	≤1	2	2	4	≤2	32	4
I41152	Blood	2018	F	52	4	≤1	≤1	16	≤2	4	0.25	≤1	2	8	4	≤2	8	4
I60584	Blood	2018	F	73	2	≤1	≤1	16	≤2	4	0.25	≤1	4	2	8	≤2	32	4
I97824	Urine	2019	M	88	2	≤1	8	8	≤2	16	0.25	≤1	16	2	8	≤2	8	8
I93488	Sputum	2009	M	74	32	8	8	8	≤2	16	1	≤1	16	8	8	≤2	8	8
I92986	Urine	2019	F	73	32	≤1	16	8	≤2	2	2	≤1	2	2	8	≤2	8	8
C80117	Ear	2009	F	3	16	≤1	8	8	≤2	4	0.25	≤1	2	2	4	≤2	8	4
U14706	Urine	2009	M	90	8	≤1	≤1	8	≤2	4	1	≤1	4	4	8	≤2	8	4
I202628	Sputum	2019	M	68	2	≤1	≤1	8	≤2	2	0.25	≤1	2	2	8	≤2	8	4
MIC Breakpoint					R > 16	R > 4	R > 4	R > 16	R > 8	R > 8	R > 0.5	R > 2	R > 8	R > 8	R > 16	R > 16	R > 16	R > 16

* M: male; F: female. Identification of *P. aeruginosa* clinical isolates using VITEK2, MicroScan WalkAway as well as MALDI-TOF (for strains isolated after 2017). Antibiograms performed using the Kirby–Bauer method using Müeller–Hinton agar according to CLSI rules (for strains isolated until January 2014) and according to EUCAST afterward using VITEK2 and MicroScan WalkAway. Metallo-beta-lactamases search was done using Etest (imipenem/imipenem + EDTA) and the Kirby–Bauer method using Müeller–Hinton agar. Antibiotics and concentrations tested: AK: amikacin (30 µg); TOB: tobramycin (10 µg); CN: gentamicin (30 µg); ATM: aztreonam (30 µg); FEP: cefepime (30 µg); CAZ: ceftazidime (10 µg); CIP: ciprofloxacin (5 µg); CS: colistin sulfate (25 µg); IMI: imipenem (10 µg); MRP: meropenem (10 µg); PRL: piperacillin (30 µg); TZP: piperacillin + tazobactam (36 µg); TC: ticarcillin (75 µg); TTC: ticarcillin + clavulanic acid (85 µg). 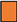 Isolates showing resistance to a particular antibiotic according to the MIC Breakpoint (EUCAST, http://www.eucast.org/clinical_breakpoints/, accessed 20 January 2020).

**Table 2:** Lytic spectra against *P. aeruginosa* PAO1 and different clinical isolates.

Isolation Source	Phage	Bacterial Isolates																				
		PAO1	I500546	1499131	C364224	U570696	U572569	I66897	I29074	I41151	H73832	U88885	I60026	I51359	I60584	I97824	I93488	I92986	C80117	U14706	I202628	Efficacy (%) *
SPC (Microgen)	SPCA																					55
	SPCB																					55
	SPCC																					45
	SPCE																					45
	SPCF																					55
	SPCG																					50
Sewage (2013)	SMS9																					45
	SMS10																					30
	SMS11																					20
Sewage (2019)	SMS12																					35
	SMS13																					30
	SMS14																					45
	SMS15																					30
	SMS16																					55
	SMS17																					30
	SMS18																					20
	SMS19																					35
	SMS20																					40
	SMS21																					30
	SMS22																					45
	SMS23																					30
	SMS24																					35
	SMS25																					30
	SMS26																					40
	SMS27																					15
	SMS28																					45
	SMS29																					45
	SMS30																					45

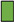
 lysis from within; 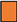 lysis from without; 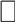 no lysis detected; 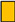 phages selected; * percentage of isolates that are susceptible to a given phage.

**Table 3:** Susceptibility of *P. aeruginosa* surviving cells recovered after 24 h from the different time–kill experiments with different phage combinations.

	Susceptibility Profile					Susceptibility of Surviving Cells (%)												
Pattern	SPCB	SPCG	SMS12	SMS21	SMS29	Control	5PCF	4PCF	3PCF	SPCB + SPCG	SMS12 + SMS21	SMS12 + SMS29	SMS21 + SMS29	SPCB	SPCG	SMS12	SMS21	SMS29
1	R	R	R	R	R		10.0	14.3	25.0	33.3	7.5	80.0	25.0	19.1		6.3		
2	R	R	R	R	S		5.0	28.5	25.0				12.5					
3	R	R	R	S	R		5.0	14.3		11.1								
4	R	R	R	S	S		10.0			22.2	15.0					6.2		
5	R	R	S	R	R		15.0	14.3	25.0			20.0	12.5					
6	R	R	S	R	S				12.5				12.5	4.8				
7	R	R	S	S	R					11.1	5.5		12.5			25.0		
8	R	R	S	S	S	30.0	20.0							4.8		18.8		
9	R	S	R	R	R		5.0											
10	R	S	S	R	S		15.0				10.0			9.5		18.8		
11	R	S	S	S	R			14.3										
12	R	S	S	S	S		15.0	14.3			28.0			42.9	100.0	12.5		
13	S	R	R	R	R									4.8				
14	S	R	S	R	R				12.5				12.5					
15	S	R	S	S	S	30.0				22.2			12.5	14.3				
16	S	S	S	R	S						35.0					12.5	100.0	100.0
17	S	S	S	S	S	40.0												
R *	79.6	64.7	35.3	52.9	47.0	100.0	100.0	85.7	100.0	100.0	66.5	100.0	87.5	80.9	0.0	12.5	100.0	0.0
S *	29.4	35.3	64.7	47.1	53.0	0.0	0.0	14.3	0.0	0.0	33.5	0.0	12.5	19.1	100.0	71.5	0.0	100.0

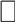
 surviving *P. aeruginosa* cells that became resistant; 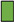 surviving cells that remained susceptible to the phage used in the treatment. When cocktail formulations were used, the value is highlighted in green only if the survivors remained susceptible to all phages present in the specific formulation. * R and S refer to the percentage of survivors showing resistance (R) or susceptibility (S) to a particular phage. 5PCF (phages SPCB + SPCG + SMS12 + SMS21 + SMS29); 4PCF (SPCG + SMS12 + SMS21 + SMS29); 3PCF (SMS12 + SMS21 + SMS29).

**Table 4:** Swimming and swarming motilities of *P. aeruginosa* cells surviving 24 h of treatment with different phage formulations.

	Swimming (%)			Swarming (%)			
Cells Surviving Specific Treatment	No	Reduced to Moderate *	Good ^†^	No	Dendritic	Smooth Edge	Suppressor
Control			100.0		100.0		
SPCB	48.6	11.4	40.0		50.0	37.5	12.5
SPCG	50.0	9.1	40.9	8.4	58.3	33.3	
SMS12	44.4	14.8	40.7	66.7		11.1	22.2
SMS21		12.5	87.5	22.2	66.7	11.1	
SMS29	20.0	10.0	70.0	62.5	25.0	12.5	
5PCF	73.7		26.3	15.8	5.2	79.0	
4PCF	38.4	30.8	30.8	57.1	42.9		
3PCF	42.9	21.4	35.7	87.5	12.5		
SPCB + SPCG	45.5	4.5	50.0		63.6	27.3	9.1
SMS12 + SMS21	71.4	14.3	14.3	71.4	28.6		
SMS12 + SMS29	55.6	33.3	11.1	28.6	14.3	57.1	
SMS21 + SMS29	60.0	26.7	13.3	25.0	50.0	25.0	

* diameter varying between 0 and 10 mm; ^†^ diameter above 10 mm; 5PCF (phages SPCB + SPCG + SMS12 + SMS21 + SMS29); 4PCF (SPCG + SMS12 + SMS21 + SMS29); 3PCF (SMS12 + SMS21 + SMS29); 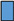 predominant swimming pattern; 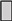 predominant swarming pattern.

## Data Availability

The data presented in this study are available on request from the corresponding author.

## Supplementary Materials

The following are available online at https://www.mdpi.com/article/10.3390/antibiotics10070877/s1, Table S1: Clusters and number of singletons formed using OrthoVenn2 by the different phages used in this work, Table S2: Percentage of identity after MAFFT alignment (65% similarity (5.0/-4.0)) and tree building using PHYML (Hasegawa-Kishino-Yano substitution model, no bootstrapping/likelihood) of phage SPCB with other homologous phages, Table S3: Percentage of identity after MAFFT alignment (65% similarity (5.0/-4.0)) and tree building using PHYML (Hasegawa-Kishino-Yano substitution model, no bootstrapping/likelihood) of phage SPCG with other homologous phages, Table S4: Percentage of identity after MAFFT alignment (65% similarity (5.0/-4.0)) and tree building using PHYML (Hasegawa-Kishino-Yano substitution model, no bootstrapping/likelihood) of phage SMS12 with other homologous phages, Table S5: Percentage of identity after MAFFT alignment (65% similarity (5.0/-4.0)) and tree building using PHYML (Hasegawa-Kishino-Yano substitution model, no bootstrapping/likelihood) of phage SMS21 with other homologous phages, Table S6: Percentage of identity after MAFFT alignment (65% similarity (5.0/-4.0)) and tree building using PHYML (Hasegawa-Kishino-Yano substitution model, no bootstrapping/likelihood) of phage SMS29 with other homologous phages, Figure S1: Progressive MAUVE whole-genome alignment (using MUSCLE 3.6) of phage SPCB (upper genome) with five Phikmvviruses based on MAFFT alignment, Figure S2: Figure S2. Progressive MAUVE whole-genome alignment (using MUSCLE 3.6) of phage SPCG (upper genome) with five Phikmvviruses based on MAFFT alignment, Figure S3: Progressive MAUVE whole-genome alignment (using MUSCLE 3.6) of phage SMS12 (upper genome) with various Pbunaviruses, Figure S4. Progressive MAUVE whole-genome alignment (using MUSCLE 3.6) of phage SMS21 (upper genome) with various Pbunaviruses, Figure S5. Progressive MAUVE whole-genome alignment (using MUSCLE 3.6) of phage SMS29 (upper genome) with various Pbunaviruses.
