# Supplementary material for: Pseudomonas aeruginosa PAO 1 In Vitro Time–Kill Kinetics Using Single Phages and Phage Formulations—Modulating Death, Adaptation, and Resistance
Source: Antibiotics (Basel). 2021 Jul 19;10(7):877. doi: 10.3390/antibiotics10070877 (PMC8300829; doi:10.3390/antibiotics10070877)
Supplement: Supplementary file 1 [file antibiotics-10-00877-s001.zip › antibiotics-1283798-supplementary.pdf]

Table S1. Clusters and number of singletons formed using OrthoVenn2 by the different phages used in this work

Phage	Proteins	Clusters	Singletons
**SPCB**	56	52	4
**SPCG**	53	52	1
**SMS12**	89	88	1
**SMS21**	88	88	0
**SMS29**	88	85	3

Table S2. Percentage of identity after MAFFT alignment (65% similarity (5.0/-4.0)) and tree building using PHYML (Hasegawa-Kishino-Yano substitution model, no bootstrapping/likelihood) of phage SPCB with other homologous phages

**Phage/Accession no.**	EU056923	**Phage SPCB**	NC_017865	LN610580	NC_005045	NC_011107	NC_028836	NC_010326	NC_022746	NC_022091	NC_027375	MH107770	NC_026602	NC_012418	NC_009935	MH979674	NC_016764	NC_042104
EU056923		**99.1**	99.1	97.8	94.8	94.1	91.3	90.7	91	88.8	89.5	88.1	88.3	87.6	84.4	61.6	35.3	34.4
**Phage SPCB**	**99.1**		**98.4**	**97.1**	**94.2**	**93.3**	91.1	90.4	90.6	88.5	89	87.8	87.9	87.6	84.2	61.2	35.2	34.3
NC_017865	99.1	**98.4**		98.3	94.2	94.2	91.5	91	91.3	89.1	89.7	88.5	88.4	88	84.2	61.9	35.3	34.3
LN610580	97.8	**97.1**	98.3		92.9	93.6	92.4	92.2	91.2	89.1	89.8	88.9	88.3	88.9	83.8	62.3	35.4	34.5
NC_005045	94.8	**94.2**	94.2	92.9		96.8	88	88.2	88.4	89.9	90.1	85.5	85.8	85.1	82.1	60	34.9	34.1
NC_011107	94.1	**93.3**	94.2	93.6	96.8		90.1	89.8	89.8	91.4	91.4	86.6	86.3	85.3	82.7	60.4	35.1	34
NC_028836	91.3	91.1	91.5	92.4	88	90.1		91.2	89.7	88.3	87.8	88.5	89.3	85.6	82.5	63.6	35.6	34.4
NC_010326	90.7	90.4	91	92.2	88.2	89.8	91.2		93.8	89.1	88.1	87.2	86.2	88.1	82.5	62	35.2	34
NC_022746	91	90.6	91.3	91.2	88.4	89.8	89.7	93.8		91.7	91.4	88.6	87.8	88.5	83.8	62.2	35.3	34.4
NC_022091	88.8	88.5	89.1	89.1	89.9	91.4	88.3	89.1	91.7		93.6	86.5	87.9	85.3	81.7	61	35	34
NC_027375	89.5	89	89.7	89.8	90.1	91.4	87.8	88.1	91.4	93.6		85.9	85.7	85.5	83.9	61.4	35.2	34.2
MH107770	88.1	87.8	88.5	88.9	85.5	86.6	88.5	87.2	88.6	86.5	85.9		86.8	85.2	81	60.9	35.7	34.2
NC_026602	88.3	87.9	88.4	88.3	85.8	86.3	89.3	86.2	87.8	87.9	85.7	86.8		85.3	83.1	61.1	35.5	34.4
NC_012418	87.6	87.6	88	88.9	85.1	85.3	85.6	88.1	88.5	85.3	85.5	85.2	85.3		82.6	59.4	35.3	34.6
NC_009935	84.4	84.2	84.2	83.8	82.1	82.7	82.5	82.5	83.8	81.7	83.9	81	83.1	82.6		59.1	34.9	33.9
MH979674	61.6	61.2	61.9	62.3	60	60.4	63.6	62	62.2	61	61.4	60.9	61.1	59.4	59.1		25.9	23.3
NC_016764	35.3	35.2	35.3	35.4	34.9	35.1	35.6	35.2	35.3	35	35.2	35.7	35.5	35.3	34.9	25.9		51.7
NC_042104	34.4	34.3	34.3	34.5	34.1	34	34.4	34	34.4	34	34.2	34.2	34.4	34.6	33.9	23.3	51.7	

In **bold,** the five hits with the highest identity percentage. In grey is highlighted the row/column representing phage SPCB

Table S3. Percentage of identity after MAFFT alignment (65% similarity (5.0/-4.0)) and tree building using PHYML (Hasegawa-Kishino-Yano substitution model, no bootstrapping/likelihood) of phage SPCG with other homologous phages

**Phage/Accession no.**	EU056923	KX711710	NC_017865	**Phage SPCG**	KU743887	NC_005045	NC_011107	KY618819	NC_022746	NC_010326	NC_022091	NC_027375	NC_028836	MH107770	NC_026602	NC_012418	NC_009935	MH979674	MG845683	NC_013638	NC_009936	NC_030923
EU056923		98.6	99.1	**96.9**	95.3	94.8	94.1	91	90.3	89.6	88.9	89.6	90.6	87.6	87.9	87	84	62	49.7	43.6	42.3	33.9
KX711710	98.6		99.5	**96.1**	94.7	94.3	94.3	91.1	90.5	89.9	89.1	89.6	90.8	88	87.8	87.2	83.8	62.3	49.6	43.6	42.3	34
NC_017865	99.1	99.5		**96.2**	94.7	94.2	94.2	91.2	90.6	89.9	89.1	89.8	90.9	88	88	87.3	83.9	62.3	49.7	43.6	42.3	34
**Phage SPCG**	**96.9**	**96.1**	**96.2**		**95.1**	**92.4**	92.3	89.3	88.8	88.2	87.3	88	90.8	87.6	87.9	85.4	83.3	62	50	43.4	42.2	33.8
KU743887	95.3	94.7	94.7	**95.1**		96.3	95.3	89.5	88.7	88.3	90.1	90.4	89.3	86.6	86.7	85.4	82.3	60.7	50	43.5	42.1	33.7
NC_005045	94.8	94.3	94.2	**92.4**	96.3		96.8	89.6	88.6	88.1	89.7	90	87.8	85.4	85.8	85	82	60.8	49.2	43.1	41.8	33.5
NC_011107	94.1	94.3	94.2	92.3	95.3	96.8		90.9	90	89.7	91.1	91.2	89.8	86.4	86.3	85.3	82.6	61.1	49.4	43.3	41.9	33.6
KY618819	91	91.1	91.2	89.3	89.5	89.6	90.9		96.4	92.5	93.3	92.5	90	88.7	87.8	88.6	83.5	63.2	49.8	44	42.2	34.1
NC_022746	90.3	90.5	90.6	88.8	88.7	88.6	90	96.4		93.8	92.2	91.9	89.2	88.2	87.4	88.1	83.4	62.7	49.9	43.9	42.3	34.1
NC_010326	89.6	89.9	89.9	88.2	88.3	88.1	89.7	92.5	93.8		89.4	88.5	90.7	86.5	85.7	87.7	82	62.5	49.3	43.4	41.9	33.7
NC_022091	88.9	89.1	89.1	87.3	90.1	89.7	91.1	93.3	92.2	89.4		93.6	88.4	86.4	87.8	85.4	81.8	61.8	49.6	43.6	41.9	33.9
NC_027375	89.6	89.6	89.8	88	90.4	90	91.2	92.5	91.9	88.5	93.6		87.9	86	85.6	85.6	83.8	62.2	49.6	43.6	42	33.9
NC_028836	90.6	90.8	90.9	90.8	89.3	87.8	89.8	90	89.2	90.7	88.4	87.9		88.6	89.4	85.5	82.4	64.5	50.4	43.6	42.2	34
MH107770	87.6	88	88	87.6	86.6	85.4	86.4	88.7	88.2	86.5	86.4	86	88.6		87	85	81.1	61.8	49.6	43.2	41.4	33.5
NC_026602	87.9	87.8	88	87.9	86.7	85.8	86.3	87.8	87.4	85.7	87.8	85.6	89.4	87		85.2	83.1	62	49.9	43.6	41.9	34
NC_012418	87	87.2	87.3	85.4	85.4	85	85.3	88.6	88.1	87.7	85.4	85.6	85.5	85	85.2		82.4	60.1	49.5	43.6	42.1	33.9
NC_009935	84	83.8	83.9	83.3	82.3	82	82.6	83.5	83.4	82	81.8	83.8	82.4	81.1	83.1	82.4		59.8	49.1	43.1	41.6	33.4
MH979674	62	62.3	62.3	62	60.7	60.8	61.1	63.2	62.7	62.5	61.8	62.2	64.5	61.8	62	60.1	59.8		37.7	25.4	22.7	19.7
MG845683	49.7	49.6	49.7	50	50	49.2	49.4	49.8	49.9	49.3	49.6	49.6	50.4	49.6	49.9	49.5	49.1	37.7		41.7	38.4	30.3
NC_013638	43.6	43.6	43.6	43.4	43.5	43.1	43.3	44	43.9	43.4	43.6	43.6	43.6	43.2	43.6	43.6	43.1	25.4	41.7		39.1	28.3
NC_009936	42.3	42.3	42.3	42.2	42.1	41.8	41.9	42.2	42.3	41.9	41.9	42	42.2	41.4	41.9	42.1	41.6	22.7	38.4	39.1		27.4
NC_030923	33.9	34	34	33.8	33.7	33.5	33.6	34.1	34.1	33.7	33.9	33.9	34	33.5	34	33.9	33.4	19.7	30.3	28.3	27.4	

In **bold,** the five hits with the highest identity percentage. In grey is highlighted the row/column representing phage SPCG.

Table S4. Percentage of identity after MAFFT alignment (65% similarity (5.0/-4.0)) and tree building using PHYML (Hasegawa-Kishino-Yano substitution model, no bootstrapping/likelihood) of phage SMS12 with other homologous phages

**Phage/Accession no.**	AP019535	MG897799	KR054028	NC_028971	KU198331	NC_011756	NC_011703	NC_017674	**Phage SMS12**	NC_028939	NC_011166	NC_041870	MK340760	NC_011165	NC_007810	NC_011810	MK318076	MN131141	MN131142	NC_026586
AP019535		92.2	86.2	87.6	34.9	35	26.9	28.5	28.6	29	35.3	35.4	35.2	36.5	26.7	26.7	31.9	31.9	33.2	32.2
MG897799	92.2		83.8	89.4	34.8	34.9	28.7	28.8	28.9	29.3	35.2	35.3	35.1	36.3	28.5	28.5	33.6	33.6	33.3	33.9
KR054028	86.2	83.8		85.2	34.7	35.1	35.1	35.1	34.9	35.1	35.3	35.2	34.9	35	34.7	34.2	35.5	35.3	35.1	35.3
NC_028971	87.6	89.4	85.2		34.8	35.3	35.3	35.3	35.1	35.2	35.5	35.3	35.1	35.1	34.9	34.4	35.7	35.5	35.3	35.5
KU198331	34.9	34.8	34.7	34.8		94.7	91.8	93.2	**92**	91.1	89.6	89.9	87.5	89.8	85.4	85	92.4	94.1	93	91.1
NC_011756	35	34.9	35.1	35.3	94.7		93.9	94.7	**94.3**	91.6	92.4	92	88.4	90.5	86.1	84.1	94.4	95.2	94.4	92.6
NC_011703	26.9	28.7	35.1	35.3	91.8	93.9		95.3	**94.5**	89.8	90.3	90.3	87.9	90.7	85.9	84.5	85	84.8	85.1	82
NC_017674	28.5	28.8	35.1	35.3	93.2	94.7	95.3		**95.4**	89.2	91.1	90.6	88.2	90.9	85	83.5	84.4	85.1	85.1	82.1
**Phage SMS12**	28.6	28.9	34.9	35.1	**92**	**94.3**	**94.5**	**95.4**		88.8	**90.5**	90.4	88	90.3	84.5	83.6	84.6	85.1	85	82.4
NC_028939	29	29.3	35.1	35.2	91.1	91.6	89.8	89.2	88.8		89.4	89.8	88.6	89.8	86	85.1	81	80.6	80.5	79.9
NC_011166	35.3	35.2	35.3	35.5	89.6	92.4	90.3	91.1	**90.5**	89.4		96.2	92.4	90.1	87	85.2	92.4	91.7	91.6	95
NC_041870	35.4	35.3	35.2	35.3	89.9	92	90.3	90.6	90.4	89.8	96.2		92.3	90.2	87	85.7	92.6	92.3	91.7	95.1
MK340760	35.2	35.1	34.9	35.1	87.5	88.4	87.9	88.2	88	88.6	92.4	92.3		89.7	89.5	88.2	89.7	88.8	88.5	92.3
NC_011165	36.5	36.3	35	35.1	89.8	90.5	90.7	90.9	90.3	89.8	90.1	90.2	89.7		88.7	88	92.3	91.4	91	92.4
NC_007810	26.7	28.5	34.7	34.9	85.4	86.1	85.9	85	84.5	86	87	87	89.5	88.7		93.3	77.9	76.8	77.1	78.6
NC_011810	26.7	28.5	34.2	34.4	85	84.1	84.5	83.5	83.6	85.1	85.2	85.7	88.2	88	93.3		76.8	76.4	76.9	77.5
MK318076	31.9	33.6	35.5	35.7	92.4	94.4	85	84.4	84.6	81	92.4	92.6	89.7	92.3	77.9	76.8		94.7	92.9	91.8
MN131141	31.9	33.6	35.3	35.5	94.1	95.2	84.8	85.1	85.1	80.6	91.7	92.3	88.8	91.4	76.8	76.4	94.7		94.3	90.9
MN131142	33.2	33.3	35.1	35.3	93	94.4	85.1	85.1	85	80.5	91.6	91.7	88.5	91	77.1	76.9	92.9	94.3		90.4
NC_026586	32.2	33.9	35.3	35.5	91.1	92.6	82	82.1	82.4	79.9	95	95.1	92.3	92.4	78.6	77.5	91.8	90.9	90.4	

In **bold,** the five hits with the highest identity percentage. In grey is highlighted the row/column representing phage SMS12.

Table S5. Percentage of identity after MAFFT alignment (65% similarity (5.0/-4.0)) and tree building using PHYML (Hasegawa-Kishino-Yano substitution model, no bootstrapping/likelihood) of phage SMS21 with other homologous phages

**Phage/Accession no.**	**Phage SMS21**	NC_017674	NC_011703	NC_011756	KU198331	NC_042079	NC_041870	NC_011166	KX171208	NC_019451	MK340760	NC_026586	MN131143	MN131141	MN131142	MK318076	MF623055	NC_042080
**Phage SMS21**		**95.4**	**94.8**	94.1	92.4	90.7	90.3	90.7	90.1	91.1	88.1	92.6	**95.6**	**95.3**	94.4	**94.5**	93.2	75.4
NC_017674	**95.4**		95.5	94.3	93.5	90.4	90.5	91.2	89.8	92	88.1	92	95.3	95.2	94.3	94.1	93	75.6
NC_011703	**94.8**	95.5		93.9	92.8	89.7	90.2	90.5	89.4	90.9	88	84	87.6	87.1	87.3	87.3	85.4	76
NC_011756	94.1	94.3	93.9		94.7	90.7	91.2	91.8	90.8	91.9	87.9	92.8	95	95	94.2	94.1	92.7	64.4
KU198331	92.4	93.5	92.8	94.7		89.8	90	89.8	88.8	91.6	87.8	91.5	93.8	93.9	92.8	92.2	91.3	62.7
NC_042079	90.7	90.4	89.7	90.7	89.8		95.2	94.3	93.3	94.2	92.1	95.8	91.4	91.6	91.9	92.4	92.6	76.4
NC_041870	90.3	90.5	90.2	91.2	90	95.2		96.2	94.4	94.6	92.3	95.2	91.8	92	91.7	92.2	92.2	77
NC_011166	90.7	91.2	90.5	91.8	89.8	94.3	96.2		95.4	95.7	92.4	95.4	91.6	91.7	91.6	92.3	92.4	76.9
KX171208	90.1	89.8	89.4	90.8	88.8	93.3	94.4	95.4		93.7	91.2	94.4	91	91.1	90.9	91.8	91.4	76
NC_019451	91.1	92	90.9	91.9	91.6	94.2	94.6	95.7	93.7		91.3	95.2	92.1	92.2	91.6	91.8	92.8	75.9
MK340760	88.1	88.1	88	87.9	87.8	92.1	92.3	92.4	91.2	91.3		92.1	88.4	88.7	88.5	89.5	89.1	74.6
NC_026586	92.6	92	84	92.8	91.5	95.8	95.2	95.4	94.4	95.2	92.1		90.7	90.7	90.2	91.5	92	75.2
MN131143	**95.6**	95.3	87.6	95	93.8	91.4	91.8	91.6	91	92.1	88.4	90.7		97.2	96.5	94	93.6	75.4
MN131141	**95.3**	95.2	87.1	95	93.9	91.6	92	91.7	91.1	92.2	88.7	90.7	97.2		94.3	94.7	92.4	75.4
MN131142	94.4	94.3	87.3	94.2	92.8	91.9	91.7	91.6	90.9	91.6	88.5	90.2	96.5	94.3		92.9	92.3	74.6
MK318076	**94.5**	94.1	87.3	94.1	92.2	92.4	92.2	92.3	91.8	91.8	89.5	91.5	94	94.7	92.9		91.6	76.6
MF623055	93.2	93	85.4	92.7	91.3	92.6	92.2	92.4	91.4	92.8	89.1	92	93.6	92.4	92.3	91.6		75.1
NC_042080	75.4	75.6	76	64.4	62.7	76.4	77	76.9	76	75.9	74.6	75.2	75.4	75.4	74.6	76.6	75.1	

In **bold,** the five hits with the highest identity percentage. In grey is highlighted the row/column representing phage SMS21.

Table S6. Percentage of identity after MAFFT alignment (65% similarity (5.0/-4.0)) and tree building using PHYML (Hasegawa-Kishino-Yano substitution model, no bootstrapping/likelihood) of phage SMS29 with other homologous phages

**Phage/Accession no.**	**Phage SMS29**	NC_017674	GU815091	NC_011703	FM897211	NC_011756	FM887021	KU198331	NC_041865	KX171208	NC_019451	KP340287	NC_011810	NC_007810	NC_026586	MN131143	MN131141	MN131142	MK318076	LT594786
**Phage SMS29**		**95.5**	**95.5**	**95**	95	94.7	94.7	92.4	90.8	90.4	91.1	91.8	84.1	85.1	92.2	**95.5**	**95.5**	94.4	94.7	93.9
NC_017674	**95.5**		100	95.3	95.3	94.7	94.7	93.2	91	89.8	92.1	91.6	84.1	85.5	92	95.2	95.1	94.2	94.1	93.9
GU815091	**95.5**	100		95.3	95.3	94.7	94.7	93.2	91	89.8	92.1	91.6	84.1	85.5	92	95.2	95.1	94.2	94.1	93.9
NC_011703	**95**	95.3	95.3		100	93.9	93.9	91.8	90.6	89.4	90.9	91	84.8	86.2	84.1	87.5	87	87.3	87.3	87.1
FM897211	95	95.3	95.3	100		93.9	93.9	91.8	90.6	89.4	90.9	91	84.8	86.2	84.1	87.5	87	87.3	87.3	87.1
NC_011756	94.7	94.7	94.7	93.9	93.9		100	94.7	92.3	91.3	92.5	93.4	84.7	86.7	92.6	95.1	95.2	94.4	94.3	94.8
FM887021	94.7	94.7	94.7	93.9	93.9	100		94.7	92.3	91.3	92.5	93.4	84.7	86.7	92.6	95.1	95.2	94.4	94.3	94.8
KU198331	92.4	93.2	93.2	91.8	91.8	94.7	94.7		89.7	88.7	91.4	91.1	85.5	86	91.1	93.9	94.1	92.9	92.3	93.9
NC_041865	90.8	91	91	90.6	90.6	92.3	92.3	89.7		95	93.6	95	86.4	88.3	94.3	91.4	91.8	91.5	92.7	92.9
KX171208	90.4	89.8	89.8	89.4	89.4	91.3	91.3	88.7	95		93.7	94.9	86.1	87.1	94.2	91	91.1	90.9	91.9	92.3
NC_019451	91.1	92.1	92.1	90.9	90.9	92.5	92.5	91.4	93.6	93.7		96	85.4	87	94.9	92.1	92.2	91.6	91.8	93.2
KP340287	91.8	91.6	91.6	91	91	93.4	93.4	91.1	95	94.9	96		86.2	87.6	95	92.5	92.6	92.1	92.1	93.4
NC_011810	84.1	84.1	84.1	84.8	84.8	84.7	84.7	85.5	86.4	86.1	85.4	86.2		93.3	79.7	78.6	78.5	79.1	79	79.7
NC_007810	85.1	85.5	85.5	86.2	86.2	86.7	86.7	86	88.3	87.1	87	87.6	93.3		80.7	79.1	79	79.3	80.1	80.5
NC_026586	92.2	92	92	84.1	84.1	92.6	92.6	91.1	94.3	94.2	94.9	95	79.7	80.7		90.9	90.9	90.4	91.7	92.7
MN131143	**95.5**	95.2	95.2	87.5	87.5	95.1	95.1	93.9	91.4	91	92.1	92.5	78.6	79.1	90.9		97.2	96.5	93.9	93.4
MN131141	**95.5**	95.1	95.1	87	87	95.2	95.2	94.1	91.8	91.1	92.2	92.6	78.5	79	90.9	97.2		94.3	94.7	93.9
MN131142	94.4	94.2	94.2	87.3	87.3	94.4	94.4	92.9	91.5	90.9	91.6	92.1	79.1	79.3	90.4	96.5	94.3		92.9	93.8
MK318076	94.7	94.1	94.1	87.3	87.3	94.3	94.3	92.3	92.7	91.9	91.8	92.1	79	80.1	91.7	93.9	94.7	92.9		94.5
LT594786	93.9	93.9	93.9	87.1	87.1	94.8	94.8	93.9	92.9	92.3	93.2	93.4	79.7	80.5	92.7	93.4	93.9	93.8	94.5	

In **bold,** the five hits with the highest identity percentage. In grey is highlighted the row/column representing phage SMS29.

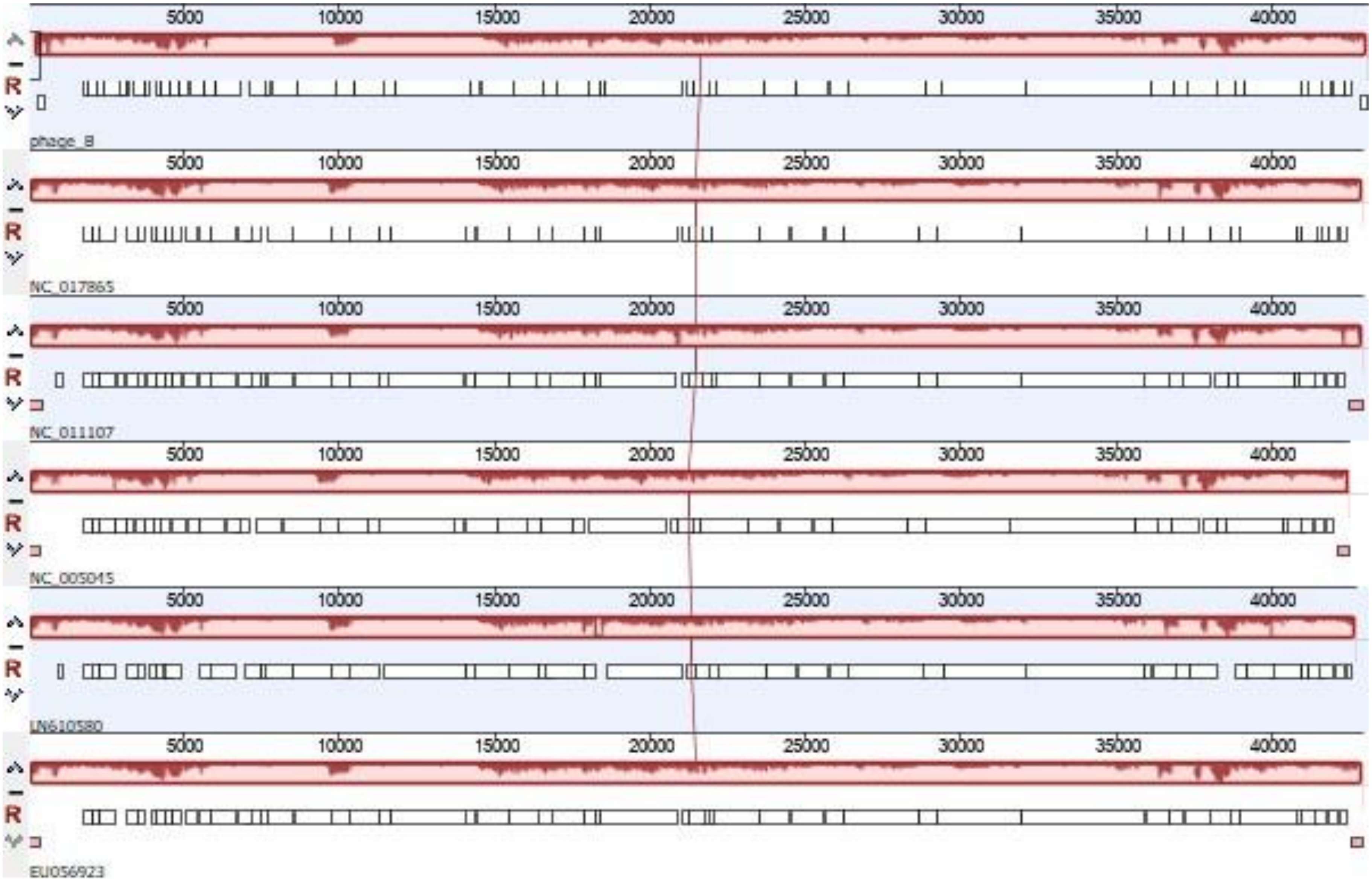

Figure S1. Progressive MAUVE whole-genome alignment (using MUSCLE 3.6) of phage SPCB (upper genome) with five Phikmvviruses based on MAFFT alignment.

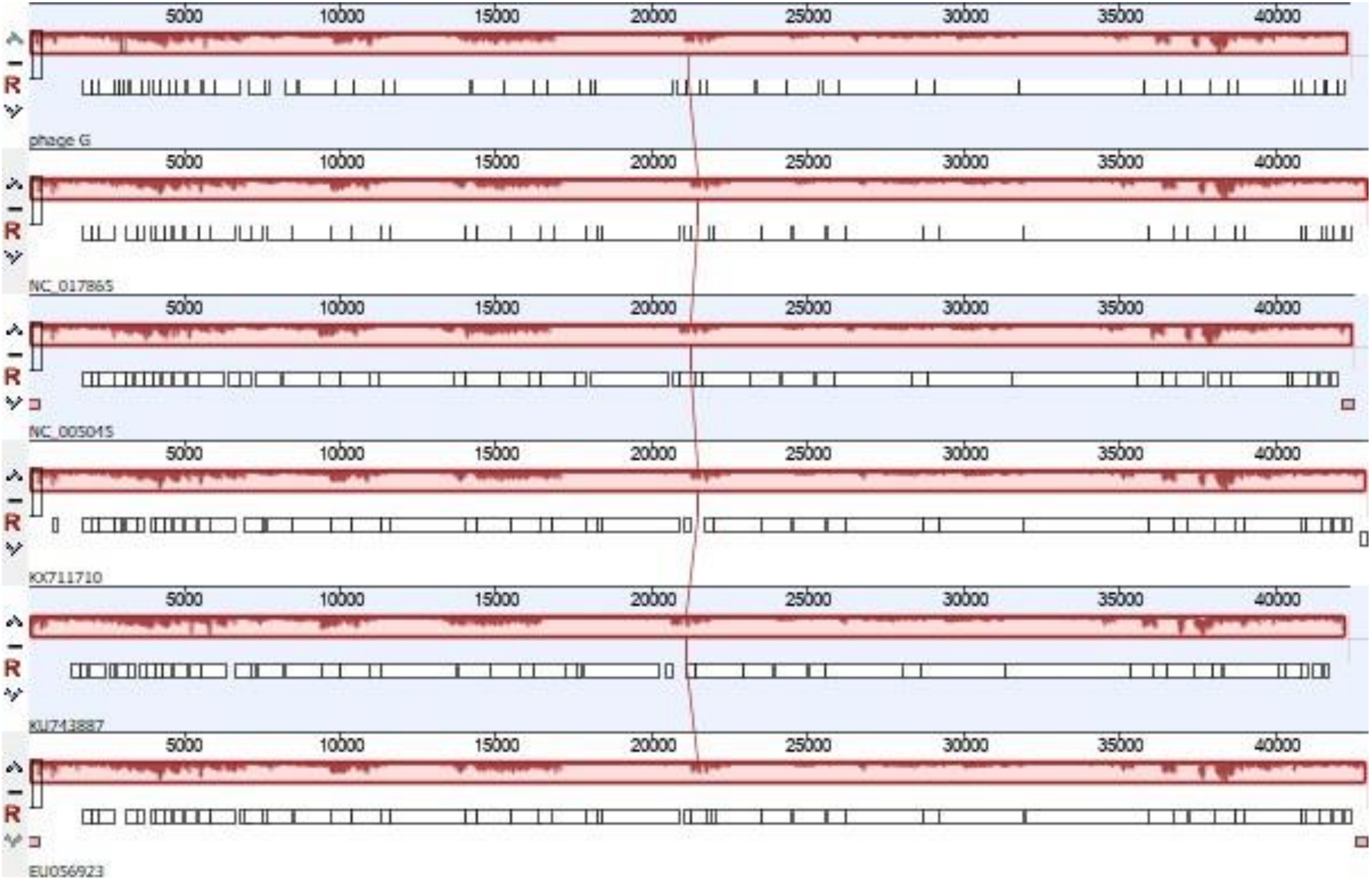

Figure S2. Progressive MAUVE whole-genome alignment (using MUSCLE 3.6) of phage SPCG (upper genome) with five Phikmvviruses based on MAFFT alignment.

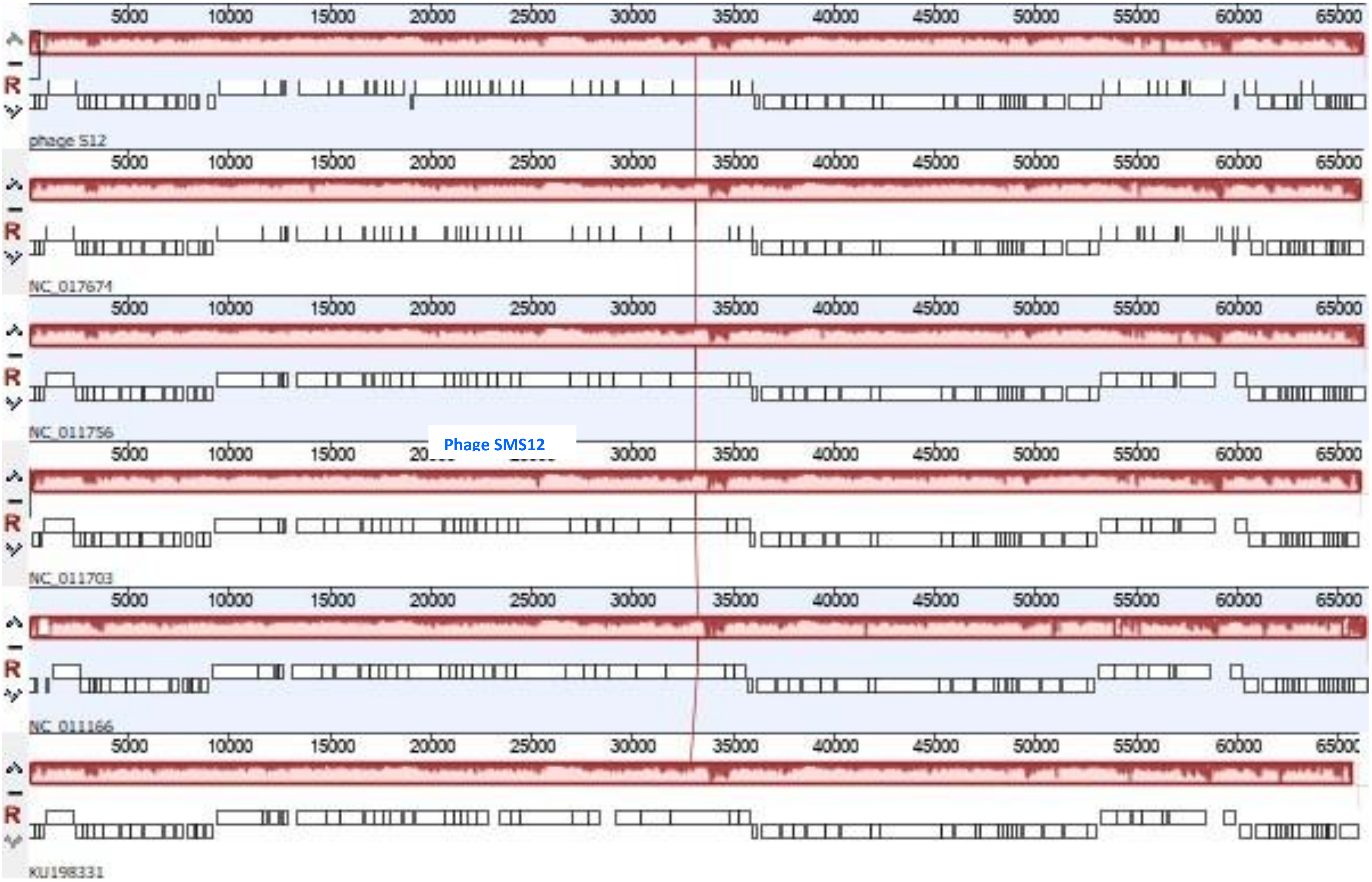

Figure S3. Progressive MAUVE whole-genome alignment (using MUSCLE 3.6) of phage SMS12 (upper genome) with various Pbunaviruses.

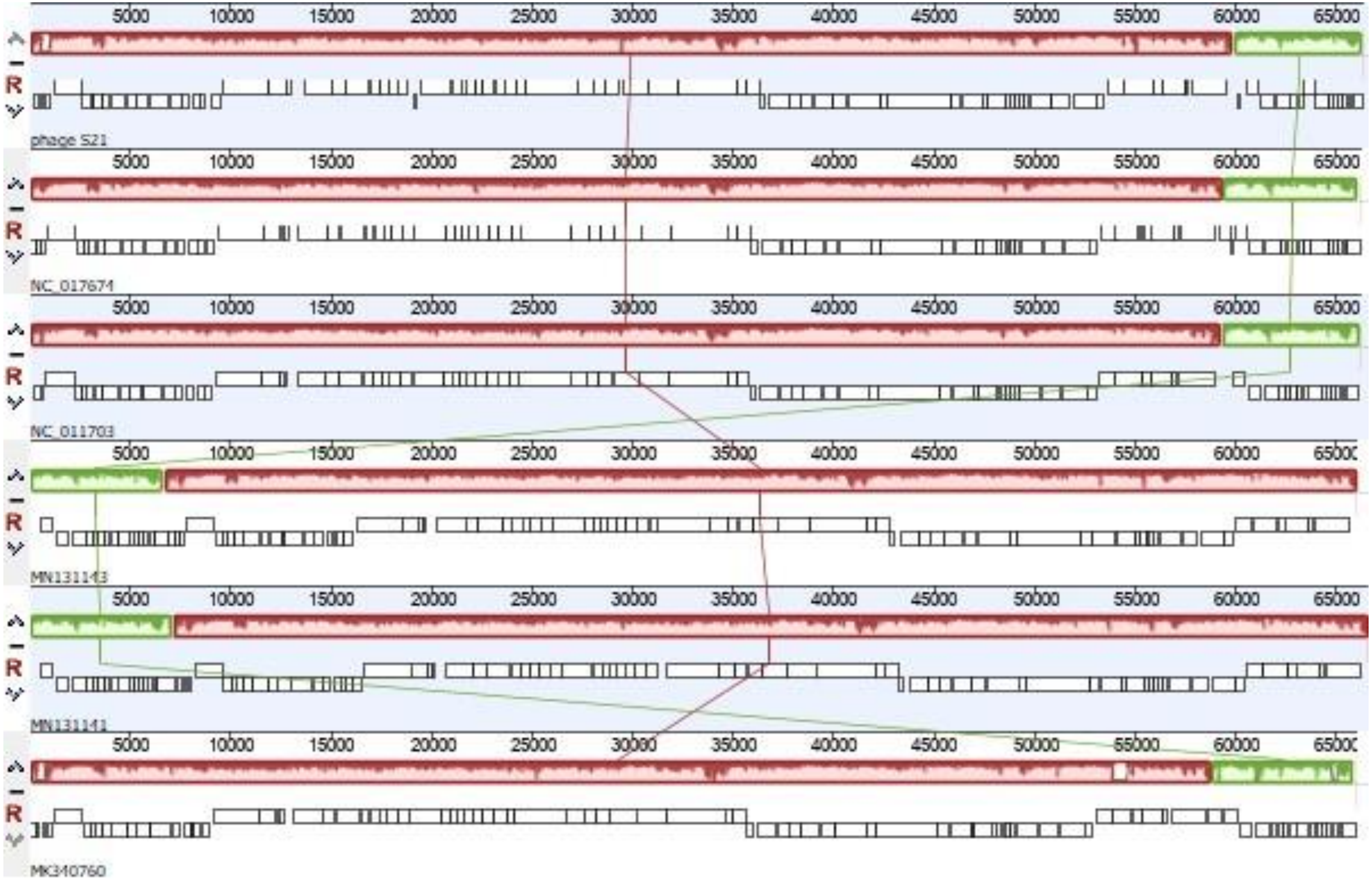

Figure S4. Progressive MAUVE whole-genome alignment (using MUSCLE 3.6) of phage SMS21 (upper genome) with various Pbunaviruses.

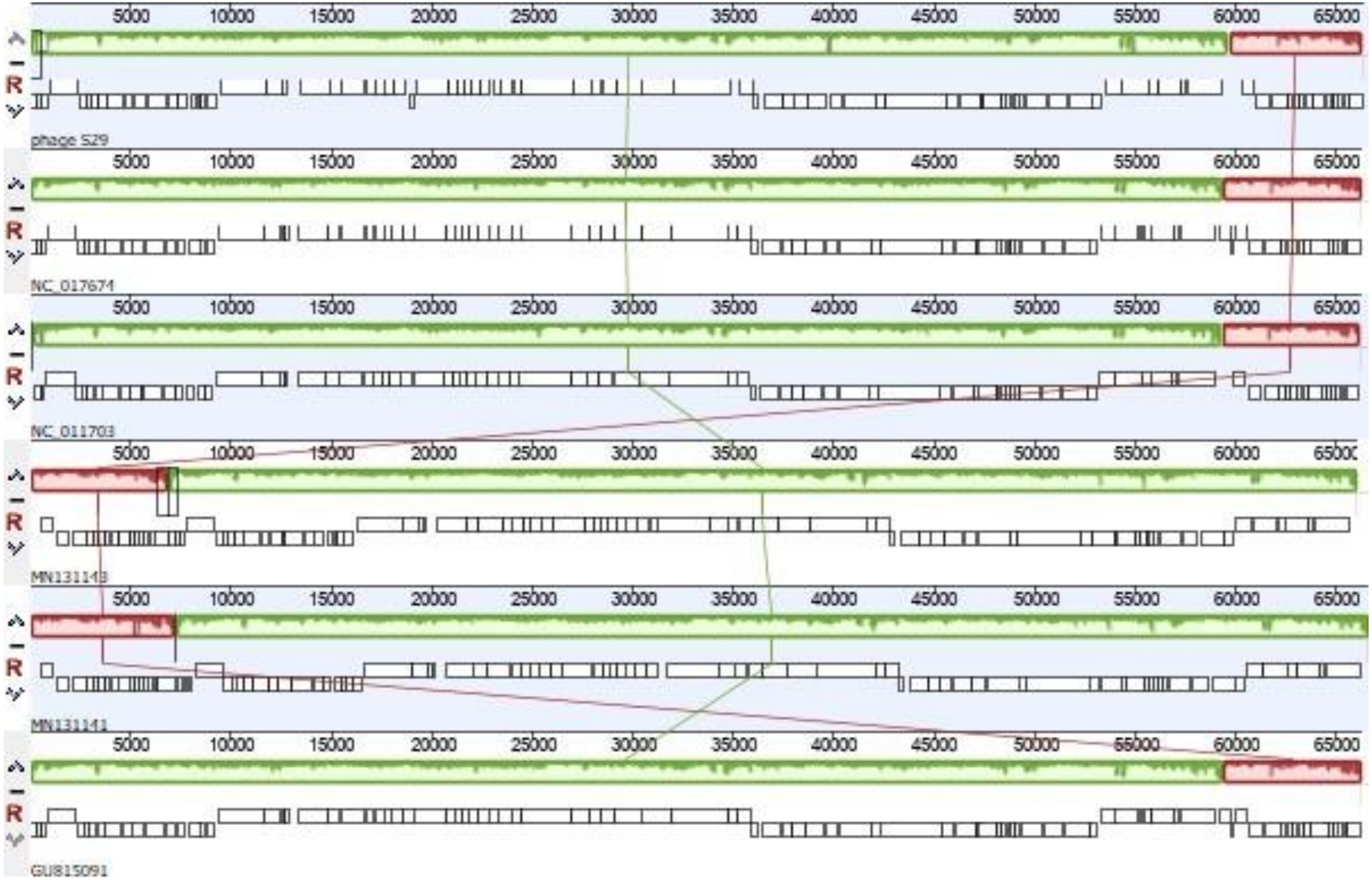

Figure S5. Progressive MAUVE whole-genome alignment (using MUSCLE 3.6) of phage SMS29 (upper genome) with various Pbunaviruses.
